# An Examination of Depression, Anxiety, and Self-Esteem in Collegiate Student-Athletes

**DOI:** 10.3390/ijerph20021211

**Published:** 2023-01-10

**Authors:** Samantha R. Weber, Zachary K. Winkelmann, Eva V. Monsma, Shawn M. Arent, Toni M. Torres-McGehee

**Affiliations:** 1 Department of Nursing and Health Science, Limestone University, Gaffney, SC 29340, USA; 2 Department of Exercise Science, University of South Carolina, Columbia, SC 29208, USA; 3 Department of Physical Education, University of South Carolina, Columbia, SC 29208, USA

**Keywords:** mental health, prevalence, behavioral health, screening

## Abstract

Mental health research exists for student-athletes in the areas of depression, anxiety, and self-esteem prevalence. However, updated prevalence rates and assessment of risks across sports, academic status, and genders are needed. Filling the gaps in research assists in the creation of patient-centered mental health screening and interventions designed for student-athletes. Therefore, the purpose is to examine the prevalence of depression, anxiety, and self-esteem in collegiate student-athletes and differences between sex, academic status, and sport type, and identify associations for risks. Using a cross-sectional design, collegiate student-athletes were surveyed to assess for risks of depression, anxiety, and self-esteem. With the use of SPSS, Chi-square analyses and multinomial logistic regressions were used. Student-athletes (22.3%) were at risk for depression, anxiety (12.5%), and low self-esteem (8%). No significant differences were found for sex, academic status, and sport type for depression or self-esteem; however, significant differences occurred for state and trait anxiety by sex. A significant association for depression and anxiety risk was found with females at risk. Depression and anxiety are present within student-athletes, regardless of sport type. Females are at a higher risk; however, all student-athletes would benefit from the creation of validated, patient-centered mental health screenings and psychotherapeutic interventions.

## 1. Introduction

There are approximately a half-million collegiate student-athletes in the National Collegiate Athletic Association (NCAA), attending over 1000 colleges and universities in over 100 athletic conferences. According to the National Institute of Mental Health, approximately 8.4% of adults aged 18 or older experienced a depressive episode, and 19.1% had an anxiety disorder in the past year [1]. More specifically, within college-age students ranging from 18–25, the prevalence was highest for depression at 17.0% and 22.3% for anxiety disorders [1]. College is considered an at-risk period for the development of mental health illnesses. According to the American College Health Association, over 30% of students reported significant signs of depression [1,2,3]. College student-athletes are a subset of the young adult population and may be at risk for stressors linked to mental health issues (e.g., disordered eating, substance or alcohol abuse). With many student-athletes participating in sport, it is reasonable to believe that numerous student-athletes participate in their sport while managing the signs, symptoms, and known risk factors of depression and anxiety. With an increase in mental health visibility, an updated examination on the prevalence of depression, anxiety, and low self-esteem for this population is warranted. 

Depression is characterized by mood changes, loss of interest or pleasure in daily activities, and the associated symptoms of sleep and eating problems, low energy, lack of concentration and self-worth [1]. With participation in sport, student-athletes are thought to be immune to mental health disorders like depression; however, research demonstrates that the general college student population and student-athletes are comparable [4,5]. Research directly investigating the prevalence and severity of depression symptoms in collegiate student-athletes varies by instruments used and by sports and sex examined. The prevalence of the risk of depression in collegiate student-athletes ranges from 15.6% to 33.2%, with first-year students and females typically reporting more symptoms [4,6,7]. When examining the depression risk prevalence in specific sports including but not limited to football, baseball, wrestling, track and field, and lacrosse, the range was from 12.1% to 35.4%, with higher rates consistent with females [8,9,10]. In the current literature, sports have been categorized as individual and team sports when examining risk of depression in collegiate student-athletes, indicating that individual sports may be at an increased risk over team sports [10,11]. With a younger population, a lower prevalence rate of 8% was found for depression and anxiety, and specifically 13% for individual sports and 7% for team sports, further supporting sport type as being associated with the risk of depression and anxiety in student-athletes [11]. The previous research on prevalence rates for team versus individual sports is based on a younger population, and there are different validated measures used throughout the research to assess for the presence of the risk of depression. 

Anxiety is commonly known as a reaction to a perceived stressful or dangerous situation that can have debilitating effects on daily activities and performance. State anxiety refers to a temporary response to a stressful advent and trait anxiety is defined as a personality feature or predisposition [12]. Athletes often experience state anxiety during situations that create pressure, for example if a free throw determines the outcome of a basketball game. However, trait anxiety refers to characteristics of a person, where an individual is anxious about general unknown outcomes. Researchers have identified that high levels of trait anxiety may lead to an increase in state anxiety during performance [13]. However, there is limited research focusing on the examination of state and trait anxiety prevalence in student-athletes. According to current literature by Li and colleagues [14], one-third of student-athletes reported anxious symptoms prior to the season beginning with a significantly higher risk for sport injury. Furthermore, previous studies examining student-athletes primarily occurred during preseason training and did not find significance for gender, sport, or academic status differences and the state and trait anxiety scores [8,14]. However, both studies by Yang et. al. [8] and Li et. al. [14], indicated that the link between depression and anxiety is associated with higher levels of pain and injury incidence. Therefore, examining anxiety prevalence rates in student-athletes by sex, academic status, and sport type and determining additional risks for depression and anxiety is warranted to help clinicians prevent additional injury. Without further research on depression and anxiety prevalence, it is difficult to develop preventative mental health programs and interventions for current conditions. 

Participation in sport facilitates positive mental health behaviors, including self-confidence, positive self-esteem, and social support [15]. Individuals with positive mental health behaviors may be utilizing their social support systems to cope and manage stress in helpful ways to lower risks for depression and anxiety. However, student-athletes may be more susceptible to mental health issues due to the demands of sport participation (e.g., sports injury, coach expectations) [9]. Student-athletes are thought to be protected from mental health issues because of increased self-esteem, a sense of connectedness, and social support from their teammates [15]. There is an established relationship between self-esteem and depression, indicating that self-esteem is associated with depression. In those with lower self-esteem, depression rates tend to be higher, whereas in student-athletes with a higher self-esteem a lower rate of depression was found [15]. However, this study is out of date, and updated research for student-athletes is needed. 

Clinicians providing medical services within the collegiate sports setting should be mindful of comorbidities of mental illnesses and which student-athletes are at the highest risk. Additionally, being able to recognize the common mental health illnesses and risks among student-athletes can help guide clinicians to utilize validated patient-centered screenings to identify those who may need referral for psychotherapeutic intervention [16]. Early screening, identification, and intervention can allow healthcare professionals to gain more information on their patients; furthermore, this allows providers to ask in-depth questions tailored to their patients’ needs, and to develop strategies (i.e., goal setting, coping mechanisms) to support their mental and physical needs [17]. Student-athletes who have an individualized approach to their needs are more likely to communicate with their providers and trust the clinician providing healthcare [18], and with a tailored approach to healthcare, specifically mental health care, student athletes are able to receive help before signs and symptoms begin to manifest. While all student-athletes have unique personal stressors and individual experiences, understanding the associations between depression, anxiety, self-esteem, sport type, and sex may help clinicians choose to use validated screenings and further develop interventions for managing symptoms for their patients. Therefore, the purpose of this study was to examine the overall prevalence of depression, anxiety, and self-esteem in NCAA Division I and II collegiate student-athletes, with a secondary purpose to examine differences between depression and anxiety risk, and low self-esteem with demographic variables such as sex, academic status (e.g., freshman, sophomore, etc.) and sport type (e.g., power, ball sports, technical, endurance, etc.); and lastly to identify associations for depression, anxiety, and low self-esteem. 

## 2. Materials and Methods

### 2.1. Participants

Participants were NCAA Division I and II student-athletes (*n* = 615; age 20 ± 1 years; males: *n* = 233, height: 184.1 ± 0.5 cm, weight: 91.5 ± 0.15 kg; females: *n* = 382, height = 168.4 ± 0.39 cm, weight: 63.25 ± 19.8 kg) from across 40 institutions. To be included in the cross-sectional study, the student-athletes had to be between the ages of 18–26 and on an active roster during the time of the survey. The Institutional Review Board approved the study, and all participants consented prior to completing the survey. The study was conducted in accordance with the Declaration of Helsinki and approved by the Institutional Review Board (or Ethics Committee) of the University of South Carolina (protocol code Pro00092702 and date of approval 1 November 2019). 

### 2.2. Instruments

#### 2.2.1. Demographic Information

The demographic information collected included age, sex, self-reported height, weight, body mass index (BMI), academic status, and sport. Academic status was defined as a first-year, sophomore, junior, or senior. Participants that were considered fifth-year seniors or graduate students were coded as seniors. Sport type was classified using prior classification by Sundgot-Borgen [19], by sorting sports into groups of endurance (i.e., cross country, track, swimming), aesthetic (i.e., cheerleading, diving, dance, equestrian), power (i.e., football), ball (i.e., baseball, softball, basketball, soccer, volleyball, beach volleyball), and technical sports (i.e., golf, tennis) [19]. 

#### 2.2.2. Center for Epidemiologic Studies Depression Scale 

The Center for Epidemiologic Studies Depression Scale (CESD) is a self-report measure of depressive symptoms. There are eight different subscales, including sadness, loss of interest, appetite, sleep, thinking/concentration, guilt, worthlessness, being tired, fatigue, movement, and suicidal ideation over the past week. Student-athletes selected how often they have felt or behaved in a particular way on a scale of 1 (rarely or none of the time) to 4 (most or all the time) during the past week. Any scores higher than 16 indicates an individual is at risk for depression [20]. The internal consistency for the CESD is r = 0.85 to 0.90, with a test-retest reliability of r = 0.45–0.7, and r = 0.91 for this study [20,21]. 

#### 2.2.3. State Trait Anxiety Inventory

The State Trait Anxiety Inventory (STAI) is a self-report tool that indicates anxiety and distinguishes between state and trait anxiety. The first 20 questions consist of statements examining how individuals feel “right now at this moment”, and individuals respond on a scale of 1 (not at all) to 4 (very much so). The second 20 questions examine how individuals generally feel on a scale of 1 (almost never) to 4 (almost always) [12,22]. State and Trait Anxiety were measured using the State-Trait Anxiety Inventory. The college student norms were used for analyses with state anxiety for females being 38.76 ± 11.96 and males at 46.47 ± 10.02, and trait anxiety for females 40.40 ± 10.15 and males 38.30 ± 9.18. For the STAI, the internal consistency coefficients range from r = 0.86 to 0.95 and the test-retest reliability ranges from r = 0.65 to 0.75 [12]. The reliability for the STAI in this study r = 0.95. 

#### 2.2.4. Rosenberg Self-Esteem Scale 

The Rosenberg Self-Esteem Scale (RSES) is one of the most widely used measures of self-esteem [23,24]. The scale consists of 10 items that are rated on a 4-point Likert scale (strongly agree = 3, agree = 2, disagree = 1, and strongly disagree = 0), and assesses how an individual thinks and feels about themselves. Each participant’s responses are summed across the 10 items and further categorized as low self-esteem or at risk (below 15) or not at risk or high self-esteem (above 15) [23,24]. The scale has been validated for college populations with a test-retest reliability ranging from 0.85 to 0.88 [23], with excellent stability and a reliability of 0.90 for this study [24].

### 2.3. Procedures

Upon receiving approval from the University of South Carolina’s Institution Review Board, participants were recruited using a snowball sampling method. Athletic trainers who worked directly with student-athletes at NCAA Division I or II intuitions were contacted with an invitation letter and a survey link and asked to forward the invitation to their current student-athletes. There was no incentive for completion of the survey. The web-based online survey (SurveyMonkey, San Mateo, CA, USA) included an invitation/consent letter, and the demographic items were followed by the CESD, STAI, and the RSES. The survey was available for 30 days, with a follow-up reminder sent to the participant every 10 days until the window closed. 

### 2.4. Statistical Analysis

We used SPSS statistical software (Version 27; SPSS Inc. Armonk, NY, USA) with an alpha set at *p* < 0.05 for all analyses. We used G*Power 3 (version 3.1.9.2., Heinrich Heine University, Dusseldorf, Germany) software to calculate power [25]. Using an alpha of 0.05 and a small effect size (0.2), our power calculation indicated that we needed a sample of 495 completed surveys to achieve an estimated power of 0.95 [25,26]. For sport type, power was estimated at 0.05 and large effect size (0.6), and our power calculation indicated a sample size of 46 per sport group to achieve an estimated power of 0.90 [25,26]. We performed basic descriptive statistics to examine the demographic information (e.g., height, weight, age, body mass index (BMI), sex, academic status, etc.) Chi-squared analyses were used to determine differences between depression, anxiety, and self-esteem risk, sex, academic status, and sport type. The results of the chi-squared tests were used to select association variables to assess each outcome using multinomial logistic regression. Education, sport, and ethnicity were not analyzed due to results from the chi-squared analyses, and sex was examined as an association variable for the outcome.

## 3. Results

A total of 821 student-athletes initiated the survey, 675 partially completed the survey, and 615 student-athletes fully completed the survey (75% completion rate). Student-athletes included in this study were from 40 institutions across 22 teams which were categorized into endurance sports (*n* = 171), aesthetic sports (*n* = 102), power sports, (*n* = 117), ball sports (*n* = 194), and technical sports (*n* = 31). Detailed demographic information can be found in Table 1. 

### 3.1. Prevalence of Depression Risk 

Overall, 22.3% (*n* = 137/615) of student-athletes were classified as being at risk for depression. A Chi-squared analysis revealed no significant differences between the CES-D and sex (Χ^2^_1,615_ = 0.00, *p* = 0.99, with females (13.8.%) reporting the same risk as males (8.5%). Please refer to Table 2 for sex distribution. No significant differences were identified for depression risk and academic status ((Χ^2^_3,615_ = 6.36, *p* = 0.095), Table 3), with sophomores (*n* = 45/154, 29.2%) and juniors (*n* = 33/149, 22.1%) reporting the highest depression risk. A Chi-squared analysis revealed no significant differences between the CES-D and sport type (Χ^2^_4,615_ = 3.427, *p* = 0.489), with ball (*n* = 48/194, 24.7%) and power (*n* = 28/117, 23.9%) sports reporting the highest risk. The distribution for CES-D risk and sport type can be found in Table 3. 

### 3.2. Prevalence of Anxiety Risk

Overall, 8.5% (*n* = 52/615) of student-athletes were classified as over the norm means for college-aged students for state anxiety, and 12.5% (*n* = 77/615) for trait anxiety. However, most of the participants were within the norms for state (66.7%, *n* = 410/615) and trait (57.4%, *n* = 353/615) anxiety, respectively. The overall raw mean scores and standard deviations by sex are presented in Table 4. A Chi-squared analysis revealed a significant difference for state anxiety and sex (Χ^2^_2,615_ = 10.46, *p* = 0.005), and for trait anxiety and sex (Χ^2^_2,615_ = 10.32, *p* = 0.006). Table 2 provides the distribution of state and trait anxiety by sex. There were no differences found for state and trait anxiety for academic status (Χ^2^_6,615_ = 3.53, *p* = 0.740), (Χ^2^_6,615_ = 4.42, *p* = 0.620) or for sport type (Χ^2^_8,615_ = 12.25, *p* = 0.141), (Χ^2^_6,158_ = 4.27, *p* = 0.832), as demonstrated in Table 5.

### 3.3. Prevalence of Low-Self Esteem 

Overall, 8.0% (*n* = 49/615) of student-athletes were classified as being at risk for low self-esteem. A Chi-squared analysis revealed no significant differences between the RSES and sex (Χ^2^_1,615_ = 1.112, *p* = 0.292), with females (7.1%) reporting a slightly lower risk than males (9.4%). The distribution for RSES by sex is found in Table 2. No significant differences were identified for low self-esteem and academic status (Χ^2^_3,615_ = 0.394, *p* = 0.942), with sophomores (*n* = 14/154, 9.1%) and juniors (*n* = 10/125, 8.0%) reporting scores indicating low self-esteem. A Chi-squared analysis revealed no significant differences between the RSES and sport type (Χ^2^_4,615_ = 4.094, *p* = 0.393), with power (*n* = 12/117, 10.3%) and endurance (*n* =17/171, 9.9%) sports reporting low self-esteem. The distribution for the RSES and sport type can be found in Table 6. 

### 3.4. Multinomial Logistic Regression

Based off of the prevalence data, the results of the multinomial analysis indicated that sex is associated with depression risk, and state and trait anxiety risk (Table 2). For depression risk, females are more likely to be at risk for depression when compared to males, with an odds ratio of 1.795 (CI: 1.184, 2.722). Females are more likely to be within the average college student mean for state anxiety as compared to males, with an odds ratio of 1.771 (CI: 1.214, 2.585) with an increase in odds by 77.1%. As for trait anxiety, females are more likely to be within or above the average college student mean for trait anxiety as compared to males, with an odds ratio of 1.427 (CI: 0.994, 2.048) and 2.539 (CI: 1.402, 4.596), respectively. 

## 4. Discussion

This study examined prevalence rates for depression, anxiety, and low self-esteem risk in a large NCAA Division I and II collegiate student-athlete sample. In addition, the differences of sex, academic status and sport were explored. This study also expands on earlier research by examining sport type classifications and investigates sex and academic status for the association with depression or anxiety risk. Furthermore, this study compares findings with previous sport categorizations and provides suggestions for future interventions. 

### 4.1. Depression 

Despite increased recognition of the importance of mental health in student-athletes, prevalence rates are still high when compared with previous literature. We found an overall prevalence of 22.3% for depression risk, similar to previous research in student-athletes using the CES-D [7,8,14]. With the risk for depression at 22.3%, nearly one in every four student-athletes report signs and symptoms of depression. Collegiate student-athletes not only have an expectation of being successful athletically but are also required to succeed personally and academically. Student-athletes are required to maintain a balance between academics and specific sport requirements, placing undue stress on the student-athletes, increasing their risk of depression. While there were no significant differences for sex, this study demonstrated that more females reported signs and symptoms of depression with the CES-D. Our findings support the suggestion that females may be at a higher risk for depression than males, further reinforcing previous efforts examining depression prevalence in student-athletes [6,7,8,9,27]. When considering the distribution of sex in the sport classification, power and ball sport groups had the highest percentage for reported symptoms. For the examination of sex with depression, significant associations were found, indicating that females are more likely to report depressive symptoms than males, which is consistent with prior research [8]. Although sport type was not found to be significant, each sport type has its own associated risks. 

It has been previously suggested that individual sports are at a higher risk for depression than team sports, and, furthermore, indicating that sport type is a risk for depression [10,11]. When examining sport in our study, we categorized our sports by the recommendations of Sundgot–Borgen [19]. With this categorization, we found no differences for sport type and depression risk; however, it is important to note that ball (basketball, soccer) and power sports (football) were the highest among sport types. Ball and power sports would fall into the category of a team sport, and while not significant, both sport groups were higher for depression risk than others that would be categorized as individual sports. The ball sport category included both females and males, while power sports only included football athletes. This finding is contrary to earlier research indicating that individual sports are more likely to report symptoms, however it should not be overlooked. The team aspect of sport is thought to be a supportive community [11], and with higher numbers from our study this could indicate possible a contention within the team community. 

In addition, no significant differences were found for academic status, indicating that the risk of depression was consistent across education levels. Contrary to previous research, first-year and sophomore students have been identified to be more at risk when compared to the other education levels [6,8]. Our results have also indicated that academic status was not a significant factor for depression risk. While the results are insignificant, it is essential to note that mental health interventions may be beneficial for all academic levels. Each academic class experiences unique individual stressors. For example, first-year students must learn to adapt to new friends, classes, and living situations while adjusting to the new team, coach, and expectations, whereas seniors are preparing to finish their athletic careers, graduate, and become working professionals in their field of study. Mental health interventions tailored to the student-athletes’ mental and physical needs such as the instruction of coping mechanisms, time management skills, and self-care may help reduce mental health prevalence among all student-athletes regardless of academic status. In addition, to ensure appropriate patient-centered care identifying and intervening with individuals that present with symptomology early can result in appropriate referrals, improving patient relationships, and outcomes. 

### 4.2. Anxiety

In collegiate student-athletes, there is limited research on the prevalence of state and trait anxiety [8,14]. Prior research has examined state and trait anxiety in student-athletes during their preseason and found no differences for sex [8,14] or collegiate class [8]. Our results are consistent with those of Yang and colleagues [8], and demonstrate that student-athletes’ state and trait scores are significantly lower than that of typical college students [22]. While student-athletes are reporting scores lower than regular college students, our results demonstrate that student-athletes are still demonstrating signs and symptoms of anxiety. In addition, when examining sex and anxiety, females are more likely to be at or above the average mean for both state and trait anxiety. It has been suggested that with an increase in trait anxiety, state anxiety scores may increase and affect performance [13]. 

In addition, no significant differences were found for state or trait anxiety scores across academic status, which is supported by Yang and colleagues [8], who revealed similar findings for anxiety scores. While not significant, it was suggested that female, freshman, or juniors had a higher tendency to report symptoms than their counterparts [8]. With increased stress to maintain academic standards, it is interesting that the scores for state anxiety were not higher. Student-athletes are required to maintain a specific grade point average each semester to maintain their scholarships and ability to participate. Similarly, a stable or general fear and worry of maintaining these requirements does not seem to be higher than that of a typical college student, which would indicate trait anxiety. Furthermore, academic status was not significant for anxiety scores in our study. Although not significant, student-athletes are still reporting anxiety symptoms. Support programs and more options for tutoring and academic success may be beneficial for all student-athletes to be successful both in the classroom and in their athletic performance.

Similar to academic status, no significant differences were found for sport type and anxiety risks. When considering state anxiety and sport, athletes would typically experience symptoms of anxiety directly after or during stressful situations [28,29]. The participants in our study were able to complete the survey at their convenience; therefore, it is possible that the student-athletes were completing the survey in a non-stressful environment and were truly not experiencing state anxiety. In the context of sport competition, state anxiety scores are known to increase during or directly after situations that are perceived as stressful. Therefore, future research in student-athletes may benefit from an examination of anxiety states throughout a competitive season. 

### 4.3. Low Self-Esteem

Low self-esteem is not a mental health disorder, but rather a behavior that can be a risk for depression and anxiety [15,30]. Individuals experiencing low self-esteem may have an inadequate perception of their performance, possibly predisposing them to mental health problems. Our study indicated that self-esteem was above the threshold for nearly all athletes sampled (92%), reflecting prior research, indicating that student-athletes have a higher sense of self-esteem when compared to non-athletes [15]. Furthermore, in a study examining college nursing students, over 70% reported low self-esteem and high academic stress [31]. From the results of our study, more student-athletes have a higher sense of self-esteem when compared to non-athletes and college nursing students. While our sample indicated a high prevalence of high self-esteem, we still had a higher prevalence for depression risk in the same sample. This contradicts previous research that demonstrates an inverse relationship with depression and self-esteem [15]. In our study, no differences were found for academic status or sport type, while previous studies did not broadly stratify participants by sport-types when examining self-esteem. Student-athletes participating in “lean” sports tend to have lower self-esteem which becomes a predictor for other mental health issues, such as eating disorders [32]. However, in our study, power and endurance athletes reported higher scores, indicating a lower self-esteem when compared to the other sport types. Endurance athletes would fall into the category of lean sports; however, power sports would not. These results demonstrate that low self-esteem occurs in both females and males, although at a low rate. While self-esteem does not seem to be a risk for student-athletes, the prevalence rates for depression and anxiety are consistent with previous research indicating that change is not occurring. 

### 4.4. Limitations and Future Research

The current findings emphasize the importance of investigating the prevalence of depression and anxiety risk in collegiate student-athletes. However, the study is not without limitations. First, it is important to note that the data is self-reported by the athletes and depends on the participants’ honest answers. Second, the self-report tools are not diagnostic, and instead indicate whether an individual is at risk. Additionally, when the data were categorized by sport, it is important to recognize that there were no females in the power sport category. We were also unable to examine the data by NCAA Division level due to the design of the survey and level of anonymity; an examination by division level may provide additional insight into risk differences. Lastly, as current mental health interventions are loosely focused on improving mental health awareness and decreasing stigma, future research should focus on creating mental health screenings from already validated screenings that can be tailored to the student-athlete population and on developing psychotherapeutic mental health interventions for student-athletes that reduces mental health symptomology and improves coping skills.

## 5. Conclusions

The present study sought to establish the prevalence of depression risk, anxiety risk, low self-esteem risk, and determine if sex, academic status, or sport type were associated. It suggests that depression and anxiety signs and symptoms are present in the student-athlete population, with females predominantly more at risk than males. Further examination into risks that may result in student-athlete mental health symptomology can help practitioners preemptively refer to mental health professionals. The student-athletes in the present sample do not appear to be at risk for low self-esteem, suggesting that other factors may be at play among the athletes that are. Furthermore, we suggest that future research create mental health screenings from validated screenings that can be tailored for the student-athlete population and the implementation of interventions to help reduce symptomology for anxiety and depression. 

## Figures and Tables

**Table 1:** Participant Demographics (Mean ± SD).

		All (*n* = 615)		Females (*n* = 382)		Males (*n* = 233)	
		*M*	*SD*	*M*	*SD*	*M*	*SD*
Age		19.6	1.3	19.5	0.1	19.7	0.1
Height (cm)		174.3	10.8	168.4	0.4	184.1	0.5
Weight (kg)		80.0	19.8	63.3	0.5	92.5	1.3
BMI (kg/cm^2^)		24.0	4.3	22.3	0.2	26.9	0.3
**Academic Status**		***%***	***n***	***%***	***n***	***%***	***n***
	Freshman	30.4	187	17.1	105	13.3	82
	Sophomore	25.0	154	16.1	99	8.9	55
	Junior	24.2	149	14.8	91	9.4	58
	Senior	20.3	125	14.1	87	6.2	38
**Sport Type**		***%***	***n***	***%***	***n***	***%***	***n***
	Endurance	27.8	171	20.5	126	7.3	45
	Aesthetic	16.6	102	16.1	99	0.5	3
	Power	19.0	117	0	0	19.0	117
	Ball	31.5	194	22.0	135	9.6	59
	Technical	5.0	31	3.6	22	1.5	9
**Ethnicity**		***%***	***n***	***%***	***n***	***%***	***n***
	African American	20.5	126	5.5	34	15.0	92
	Asian American	1.1	7	0.7	4	0.5	3
	Hispanic American	1.8	11	1.0	6	0.8	5
	Caucasian	72.2	444	51.7	318	20.5	126
	Indian/Native American	0.7	4	0.5	3	0.2	1
	“Other”	3.7	23	2.8	17	1.0	6

**Table 2:** Depression, Anxiety and Low Self-Esteem Prevalence Rates by Sex.

Sex		Depressive Symptoms (%)	Frequency (*n*)	*X* ^2^	*p*-Value
	Overall	22.3	137	0.00	0.99
	Females	13.8	85		
	Males	8.5	52		
		**State Anxiety (%)**	**Frequency (*n*)**	***X* ^2^**	***p*-Value**
	Overall	8.5	52	10.46	0.005 ^a^
	Females	7.3	28		
	Males	10.3	24		
		**Trait Anxiety (%)**	**Frequency (*n*)**	***X* ^2^**	***p*-Value**
	Overall	12.5	77	10.32	0.006 ^a^
	Females	15.2	58		
	Males	8.2	19		
		**Low Self-Esteem (%)**	**Frequency (*n*)**	***X* ^2^**	***p*-Value**
	Overall	8.0	49	1.11	0.29
	Females	7.1	27		
	Males	9.4	22		

^a^*p* Values ≤ 0.05. State and trait anxiety were measured using the State-Trait Anxiety Inventory. The college student norms were used for analyses. State anxiety means and standard deviation for females was 38.76 (11.96) and for males it was 46.47 (10.02), and trait anxiety for females was 40.40 (10.15), while for males it was 38.30 (9.18).

**Table 3:** Depression Prevalence by Academic Status and Sport.

Academic Status		Depressive Symptoms (%)	Frequency (*n*)	*X^2^*	*p*-Value
	Overall	22.3	137	6.36	0.10
	Freshman	19.3	36		
	Sophomore	29.2	45		
	Junior	22.1	33		
	Senior	18.4	23		
**Sport**		**Depressive Symptoms (%)**	**Frequency (*n*)**	***X^2^***	***p*-Value**
	Overall	22.3	137	3.43	0.49
	Endurance	22.2	38		
	Aesthetic	15.7	16		
	Power	23.9	28		
	Ball	24.7	48		
	Technical	22.6	7		

**Table 4:** State and Trait Anxiety Raw Scores. [M (SD)].

Sex	State Anxiety	Trait Anxiety
Overall	34.25 (10.13)	25.78 (9.42)
Females	35.34 (10.13)	36.73 (9.50)
Males	32.49 (9.88)	34.15 (9.05)

State and trait anxiety were measured using the State-Trait Anxiety Inventory. The college student norms were used for analyses. State anxiety means and standard deviation for females was 38.76 (11.96) and for males it was 46.47 (10.02), and trait anxiety for females was 40.40 (10.15), while for males it was 38.30 (9.18).

**Table 5:** Anxiety Prevalence by Academic Status and Sport.

Academic Status		State Anxiety (%)	Frequency (*n*)	*X* ^2^	*p*-Value
	Overall	8.5	52	3.53	0.74
	Freshman	8.6	16		
	Sophomore	9.1	14		
	Junior	10.1	15		
	Senior	5.6	7		
		**Trait Anxiety (%)**	**Frequency (*n*)**	***X* ^2^**	***p*-Value**
	Overall	12.5	77	4.42	0.62
	Freshman	13.4	25		
	Sophomore	14.9	23		
	Junior	12.1	18		
	Senior	8.8	11		
**Sport**		**State Anxiety (%)**	**Frequency (*n*)**	***X* ^2^**	***p* = Value**
	Overall	8.5	52	12.25	0.14
	Endurance	14.0	24		
	Aesthetic	8.8	9		
	Power	4.3	5		
	Ball	6.7	13		
	Technical	3.2	1		
		**Trait Anxiety (%)**	**Frequency (*n*)**	***X* ^2^**	***p*-Value**
	Overall	12.5	77	4.27	0.83
	Endurance	15.8	27		
	Aesthetic	8.8	9		
	Power	11.1	13		
	Ball	12.9	25		
	Technical	9.7	3		

**Table 6:** Low Self-Esteem Prevalence by Academic Status and Sport.

Academic Status		Low Self-Esteem (%)	Frequency (*n*)	*X* ^2^	*p*-Value
	Overall	8.0	49	0.39	0.94
	Freshman	7.5	14		
	Sophomore	9.1	14		
	Junior	7.4	11		
	Senior	8.0	10		
**Sport**		**Low Self-Esteem (%)**	**Frequency (*n*)**	***X* ^2^**	***p*-Value**
	Overall	8.0	49	4.10	0.39
	Endurance	9.9	17		
	Aesthetic	7.8	8		
	Power	10.3	12		
	Ball	5.7	11		
	Technical	3.2	1		

## Data Availability

Not available based on Institutional Review Board policies.
